# A pilot morphometric analysis of lumbar vertebrae to identify fluoroscopic biomarkers

**DOI:** 10.1016/j.inpm.2026.100748

**Published:** 2026-03-04

**Authors:** John Tran, Elysia Chau, Melissa Calleja, Roger Leekam, Eldon Loh

**Affiliations:** a Division of Anatomy, Department of Surgery, University of Toronto, Toronto, Canada; b Department of Physical Medicine and Rehabilitation, Western University, London, Canada; c Parkwood Institute Research, Lawson Research Institute, London, Canada

**Keywords:** Anatomy, Lumbar, Fluoroscopy, Osteology, Morphometry

## Abstract

**Introduction:**

Variability in lumbar vertebral anatomy and clinical outcomes necessitate a more patient-specific approach to perform lumbar medial branch radiofrequency neurotomy (LMBRFN). To identify patient-specific fluoroscopic biomarkers, a comprehensive understanding of 3D bony anatomy and its fluoroscopic appearance is required. The objective of this first-of-its-kind pilot morphometric study was to use 3D modeling technology to evaluate these correlations and identify fluoroscopic biomarkers that may distinguish optimal parallel cannula placement for LMBRFN.

**Methods:**

The lumbar vertebrae and sacra (n = 60) were photographed, reconstructed in 3D and imaged with fluoroscopy. Individual 3D model of the lumbar vertebra and sacrum were imported into Blender3D and custom code was used to simulate fluoroscopic imaging for correlational morphometric analysis with photos and radiographs. Virtual cannulae were placed parallel to the lateral necks of the superior articular processes of each 3D model to determine the cannula angle classification and identify the fluoroscopic biomarker.

**Results:**

A total of 85 out of 118 simulated cannula placements (72.0%) were classified as parasagittal (<15-deg) and 33 (28.0%) were traditional (>15-deg). Qualitative analysis of the parasagittal vs traditional vertebrae found a potential differentiating fluoroscopic biomarker based on morphometric parameters. In the 85 cases of simulated cannula placement with parasagittal trajectory, 70 (82.0%) had this fluoroscopic biomarker. For the 33 cases with traditional cannula trajectory, only 10 (30.0%) had the fluoroscopic biomarker.

**Conclusions:**

This morphometric analysis study demonstrates that 3D modelling with fluoroscopic imaging analysis is feasible to identify fluoroscopic biomarkers. Future clinical and morphometric studies are needed to develop robust fluoroscopic biomarkers to advance interventional pain medicine towards a personalized medicine paradigm based on a patient's specific anatomy.

## 1 Introduction

Fluoroscopy-guided lumbar medial branch (LMB) radiofrequency neurotomy (RFN) is commonly used to manage facetogenic low back pain, although consistent clinical benefits remain elusive [1]. One factor that may limit clinical outcomes following LMBRFN is the length of LMB nerve captured during RFN. Optimal cannula-to-nerve contact, in order to maximize the length of the LMB captured, is postulated to improve the quality and duration of pain relief following LMBRFN [2]. Historically, a standardized, one-size-fits-all approach to LMBRFN has been the standard of practice. For parallel placement along the LMB, two distinct approaches have been described. The traditional technique utilized an approximately 20-degree angulation away from the sagittal plane [3]. More recently, the use of current 3D technologies in anatomical studies has expanded the understanding of LMB anatomy, leading to the description of a parasagittal technique for parallel placement [[4], [5], [6], [7], [8], [9], [10], [11]].

Variability in lumbar vertebral anatomy, however, may necessitate a more patient-specific approach [7,8]. That is, rather than relying on a single way of placing radiofrequency cannulas along the LMB in all patients (which could be a traditional or parasagittal technique), an individual patient's anatomy may dictate the approach that is ideal. Identification of specific fluoroscopic biomarkers may allow clinicians to utilize the appearance of lumbar vertebra under fluoroscopy to determine the cannula angles (traditional or parasagittal) that are preferable for maximizing LMB capture.

To develop fluoroscopic biomarkers for LMBRFN, a comprehensive understanding of 3D bony anatomy and its appearance on 2D fluoroscopic imaging is required. No study to date has investigated correlations between 3D bony anatomy, 2D fluoroscopy images, and cannula angles. The objective of this first-of-its-kind pilot morphometric study was to use high-fidelity 3D modeling technology to evaluate these correlations, in order to develop fluoroscopic biomarkers that may distinguish optimal parallel cannula placement trajectories for LMBRFN.

## 2 Material and methods

### 2.1 Bony lumbar vertebrae procurement and photography

Twenty lumbosacral bony spine specimens (60 vertebrae) were procured from the osteology collection in the Division of Anatomy at the University of Toronto. Spines with significant damage to the superior articular and/or transverse processes were excluded from the study. Lumbar and sacral vertebrae were inspected and sorted into their respective levels (L1-Sacrum) for subsequent data collection and analysis. This osteological study was approved by the University of Toronto Health Sciences Research Ethics Board protocol #27210.

Each vertebra was individually photographed from different perspectives including posterior (AP, 0-degrees) and oblique (OBL, 20- and 30-degrees) views. To obtain photographs of each vertebra at specific angles, a protractor was used as a visual guide (Fig. 1A). Each vertebra was stabilized on a clay ball and was aligned so that the direct AP and direct L views aligned with the 0- and 180-degree lines on the protractor, respectively. This protocol enabled subsequent correlation with fluoroscopic images taken using true AP segmental imaging (i.e., squared superior vertebral endplate and midline spinous process) and 2D renderings of the high-fidelity 3D reconstruction of each vertebra (Fig. 1) [12].

**Fig. 1:**
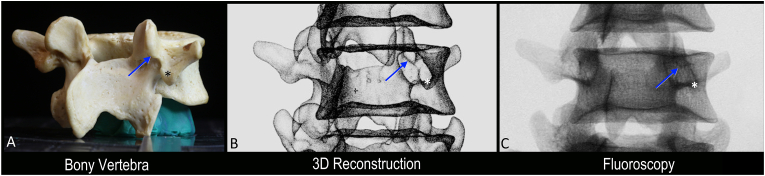
**Bony anatomy, 3D reconstruction, and fluoroscopic imaging correlation methodology.** A. Photograph of bony vertebra. B. 3D reconstruction with simulation of fluoroscopic imaging. C. Real fluoroscopic image of vertebra in A. Asterisk indicates accessory process; Blue arrow, inferior margin of the mammillary process. (For interpretation of the references to colour in this figure legend, the reader is referred to the Web version of this article.)

### 2.2 High-fidelity 3D reconstruction, fluoroscopic imaging simulation, and virtual cannula placement

High-fidelity laser scans of each vertebra were completed using a Faro Laser ScanArm (FARO Technologies Inc., USA; accuracy 0.02 mm) to capture the external surface geometry as 3D Cartesian data points. The scanned data points were manually cleaned and processed to remove scanning artifacts prior to 3D reconstruction. A corresponding high-fidelity 3D model was generated for each vertebra. Custom plugins/computer code used in conjunction with Blender3D (an open-sourced 3D modelling software) was applied to each 3D model to simulate fluoroscopic imaging appearance (Fig. 1B). Simulated fluoroscopic images were necessary to create 2D renderings of each vertebra in true AP and OBL views and enable more accurate comparison of geometry with real fluoroscopic imaging of the vertebral bones. Following the high-fidelity 3D reconstruction and fluoroscopic imaging simulation, cylinders (representing RFN cannulas) were virtually created and positioned along the lateral neck of SAP [11]. Optimal virtual cannula placement was achieved by placing the virtual cannula along the lateral neck where the greatest cannula-to-bone contact occurred, directly over the location of the medial branch. The optimally placed virtual cannula and high-fidelity 3D models were subsequently used to measure angles and classify the geometry of the lateral neck for each vertebra as traditional or parasagittal oriented.

### 2.3 Cannula angle quantification and vertebrae classification

High-fidelity 3D reconstructed vertebrae with virtual cannula placement parallel to the lateral neck of the SAP were individually rendered into 2D images. The 2D renderings were used to quantify cannula angles in the AP view. The cannula angle quantification methodology has been previously published [7,8]. Following the quantification of cannula angles in the AP view, each vertebra was grouped based on the geometry of the lateral neck where 15-degree was used as the cut off to distinguish which vertebra had a traditional, or parasagittal orientation [8]. More complex grouping of cannula angle ranges (i.e. intervals of 5-degree) was not feasible and beyond the scope of this first-of-its-kind pilot morphometric study. Supplementary figures have been published with this study for interested third parties.

### 2.4 Fluoroscopy and qualitative identification of morphometric/fluoroscopic biomarkers

Following laser 3D reconstruction, the vertebrae were assembled to create articulated lumbosacral spines and then imaged with fluoroscopy. The individual vertebra was held together using glue applied to the inferior and superior surfaces of adjacent vertebral bodies to produce fluoroscopic images like those seen in the clinical setting. Each articulated lumbosacral spine was manually rotated to capture live fluoroscopic images. Imaging data (photographic, 3D models, and radiographic images) were reviewed and qualitative assessment was performed to correlate anatomical landmarks with their corresponding fluoroscopic features (Fig. 1C). Distinct morphometric/fluoroscopic biomarker (i.e. morphology of the superior articular process/mamillary process) that distinguished different vertebra classifications (i.e. traditional vs parasagittal orientation) were identified and documented.

## 3 Results

Twenty bony spine specimens were sourced from the osteological collection in the Division of Anatomy at the University of Toronto. Following inspection of the specimens, 10 spines were excluded from the study due to substantial damage to either the superior articular and/or transverse processes in the lumbar vertebrae (i.e. missing mammillary process). A total of 10 bony spine specimens were included in this study. The lumbar vertebrae and sacra of each bony spine specimen were photographed individually (n = 60). For each bone, 7 photographs were taken (90-, 30-, 20-, 0-, 20-, 30-, 90-degee views) producing 420 images (Fig. 2 and see supplementary). Each bone (n = 60) was laser scanned to generate high-fidelity 3D models. Data for the reconstructed model of one lumbar vertebra was corrupted and was excluded from the analysis. Each 3D model (n = 59) was used to generate a simulated fluoroscopic image. Images matching the photographs were created totalling 413 images (Fig. 2 and see supplementary). Each bony spine specimen was then imaged with fluoroscopy.

**Fig. 2:**
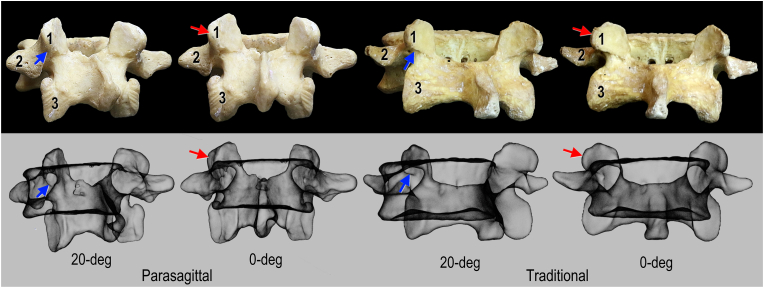
**Bony anatomy and simulated fluoroscopic imaging correlation.** Blue arrow indicates inferior margin of the mammillary process, red arrow, lateral aspect of mammillary process; 1, mammillary process; 2, transverse process; 3, inferior articular process. (For interpretation of the references to colour in this figure legend, the reader is referred to the Web version of this article.)

Optimal virtual cannula placement was then performed on each reconstructed vertebra. A total of 118 simulated cannulas were placed parallel to the lateral neck of the SAP (Fig. 3 and see supplementary). A total of 85 out of 118 simulated cannula placements (72.0%) were classified as parasagittal (<15-deg) and 33 (28.0%) were traditional (>15-deg). Qualitative analysis of the parasagittal vs traditional vertebrae found a potential differentiating fluoroscopic biomarker based on morphometric parameters (Fig. 4); specifically, positioning of the mammillary process (Fig. 4, red arrow) within the lateral boundary of the vertebral body on true AP view (Fig. 4B, green line). In the 85 cases of simulated cannula placement with parasagittal trajectory (<15-deg), 70 (82.0%) had this fluoroscopic biomarker (Fig. 4A and B). For the 33 cases with traditional cannula trajectory (>15-deg), only 10 (30.0%) had this fluoroscopic biomarker. In 23 of the 33 traditional cases the mammillary process projected laterally beyond the boundaries of the vertebral body (Fig. 4C and D).

**Fig. 3:**
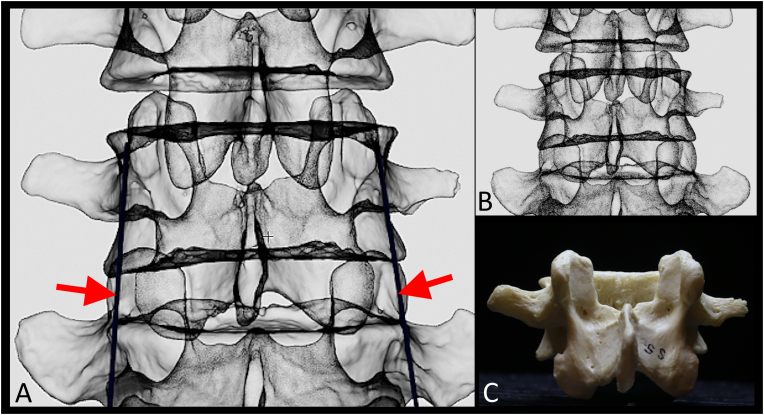
**Simulation of cannula placement for vertebra classification.** A. Simulated cannula placement with parasagittal trajectory (red arrow). B. 3D reconstructed L3 vertebra without simulated cannula placement. C. Bony vertebra. (For interpretation of the references to colour in this figure legend, the reader is referred to the Web version of this article.)

**Fig. 4:**
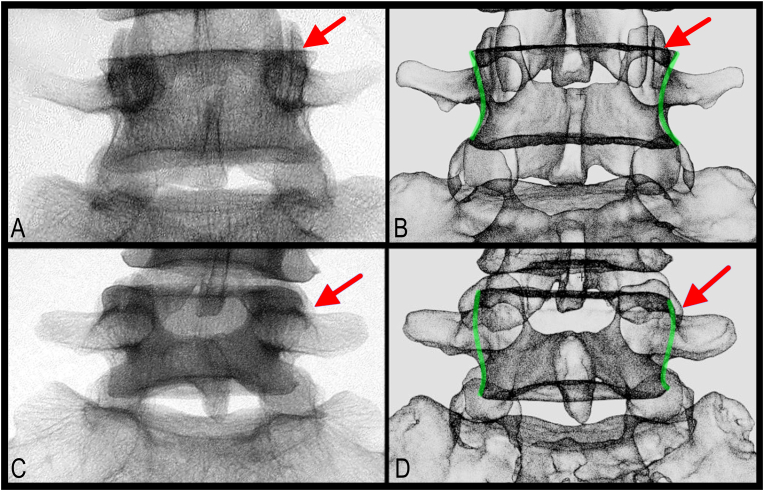
**Identification of fluoroscopic biomarker by comparing fluoroscopic image with simulated fluoroscopic imaging applied to 3D reconstructed bony vertebra.** A. Fluoroscopic image of L5 vertebra with parasagittal fluoroscopic biomarker. B. Simulated fluoroscopic imaging of 3D reconstructed L5 vertebra in panel A. C. Fluoroscopic image of L5 vertebra without parasagittal fluoroscopic biomarker. D. Simulated fluoroscopic imaging of 3D reconstructed L5 vertebra in panel C. Red arrow indicates lateral aspects of the mammillary process; green line, lateral boundary of vertebral body. (For interpretation of the references to colour in this figure legend, the reader is referred to the Web version of this article.)

## 4 Discussion

Anatomical variability of the lateral neck of SAP may account for the inconsistency in pain relief after LMBRFN [[13], [14], [15], [16], [17]]. That is, when utilizing a single, “one-size-fits-all” approach to treat a patient population with anatomical variability, clinical outcomes may be variable. A personalized approach to optimize parallel placement specific to the patient's anatomy may therefore lead to more consistent pain relief outcomes. An evidence-based method to classify these anatomic variations is necessary to develop such a personalized approach.

Based the current study, 82% of the vertebrae that have an optimal geometry for parasagittal cannula placement were observed to have a specific fluoroscopic biomarker seen in the posterior (AP) view (Fig. 4B). That is, the mammillary process was located within the lateral boundary of the vertebral body on the posterior (AP) view. Anatomically, this biomarker would make sense; since the orientation of the lateral neck is more parasagittal (i.e. parallel with the spine) the mammillary process would project more directly posterior and would therefore be more commonly located within the lateral boundary of the vertebral body (Fig. 4A and B). In contrast, if the lateral neck was at an oblique angle (i.e. optimal for traditional cannula trajectory) the mammillary process would be directed posterolateral, such that it is more likely to extend beyond the lateral boundary of the vertebral body (Fig. 4C and D). This suggests there is a higher chance of achieving parallel placement using a parasagittal approach when this particular fluoroscopic biomarker is observed. Future study is necessary to assess the clinical outcomes of this postulation. Moreover, as a pilot study, additional robust fluoroscopic biomarkers may exist and should be further investigated.

This pilot study is limited by two cannula angle groupings and identification of only a single fluoroscopic biomarker due to lack of available resources and the labor-intensive nature of this work. However, the body of data collected in this study is evidence that this methodology is feasible. Future research is needed to refine and identify more robust fluoroscopic biomarkers to progress the field of interventional pain medicine towards a personalized, anatomy-specific approach to selecting cannula trajectories when treating patients using LMBRFN.

## 5 Conclusions

This first-of-its-kind morphometric analysis study demonstrates that the high-fidelity 3D modelling methodology combined with fluoroscopic imaging analysis is feasible to identify fluoroscopic biomarkers. The fluoroscopic biomarker may be able to distinguish different lumbar vertebrae based on unique morphometric parameters which could inform cannula placement approaches. Future clinical research and further analysis to identify more robust fluoroscopic biomarkers are needed to advance the field of interventional pain medicine towards a personalized medicine paradigm based on a patient's specific anatomy.

## Conflict of interests

JT & EL have received research grants from International Pain and Spine Intervention Society (IPSIS), Avanos Medical Inc., and FUSmobile Inc. (paid directly to Lawson Research Institute). JT is a consultant with Brixton Biosciences Inc. and Merz Therapeutics (relationship ended). EL is a consultant with Brixton Biosciences Inc. The other authors have nothing to disclose.

## Figures and Tables

**figs1:**
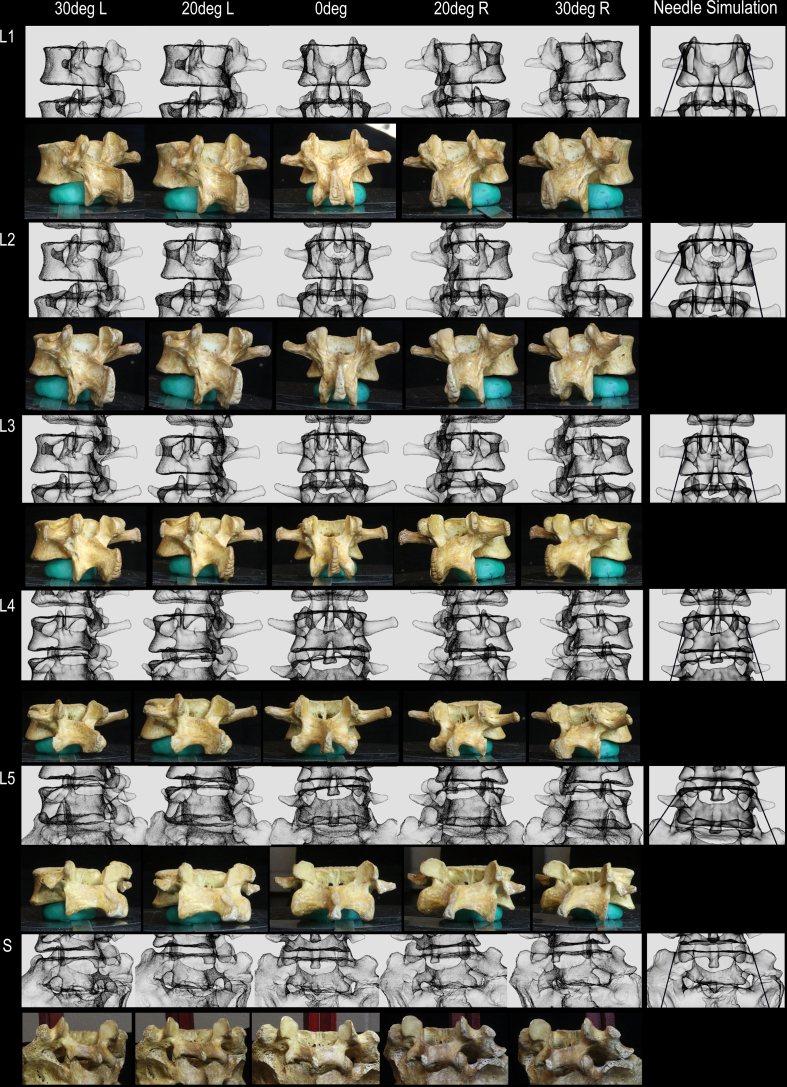


**figs2:**
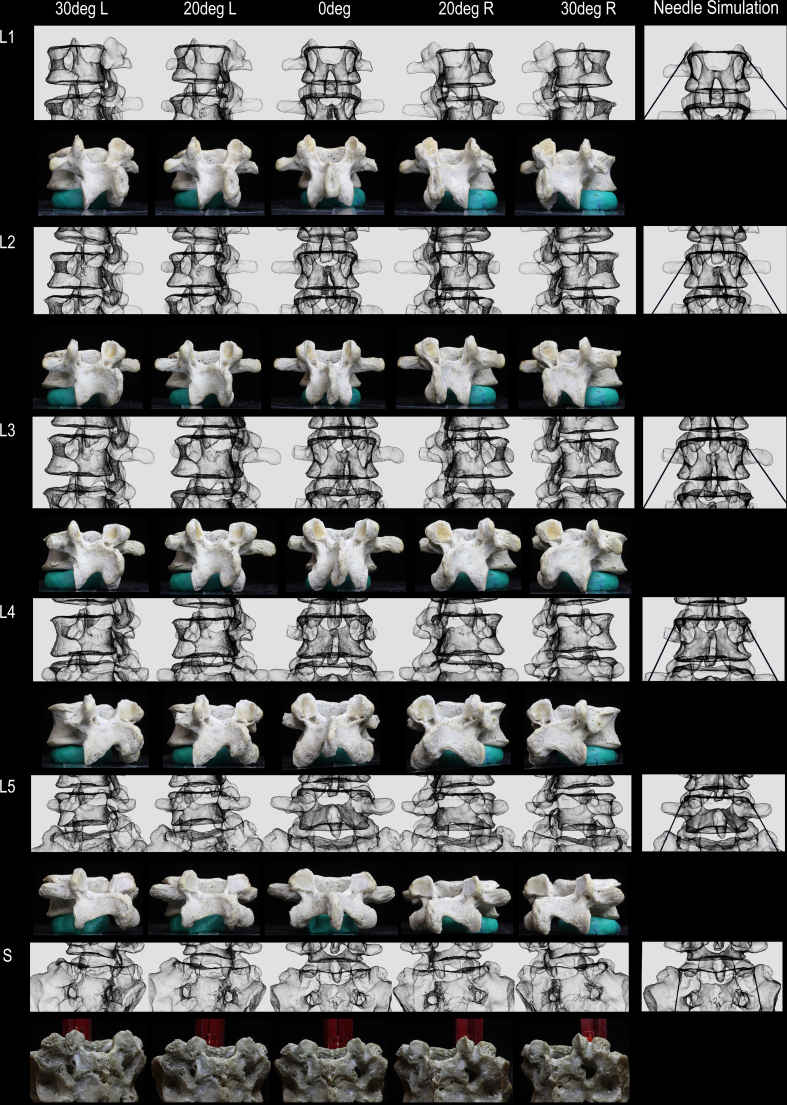


**figs3:**
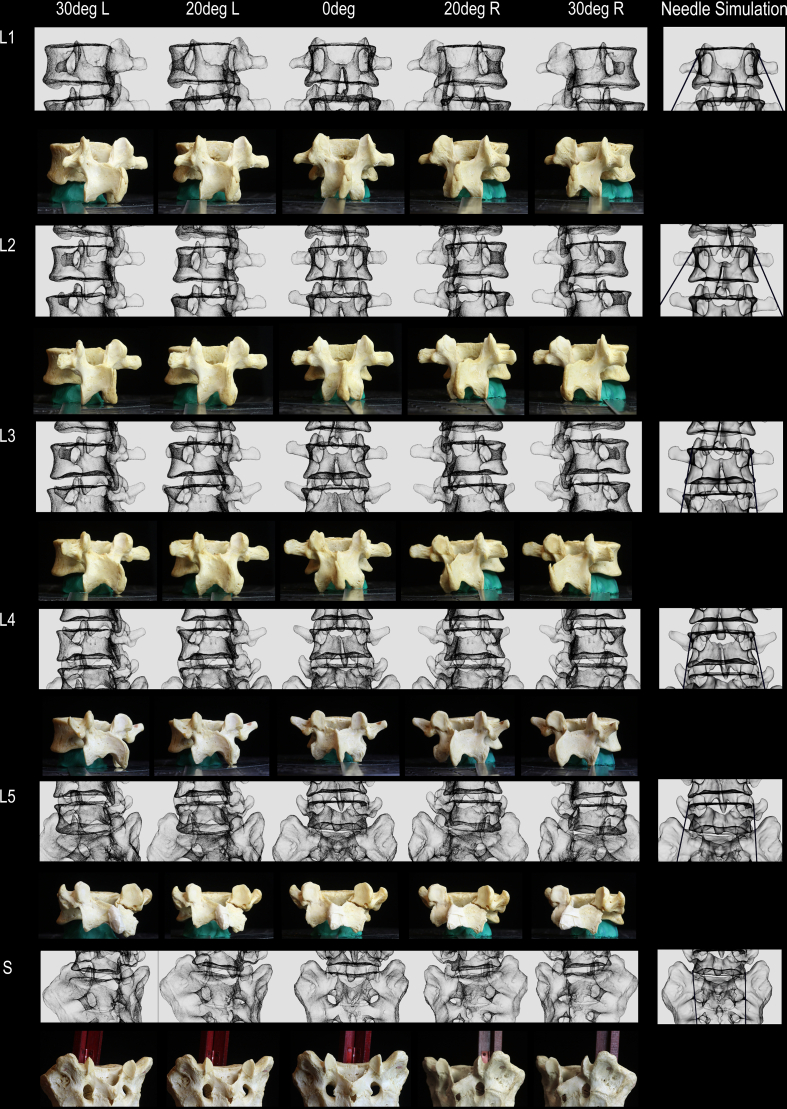


**figs4:**
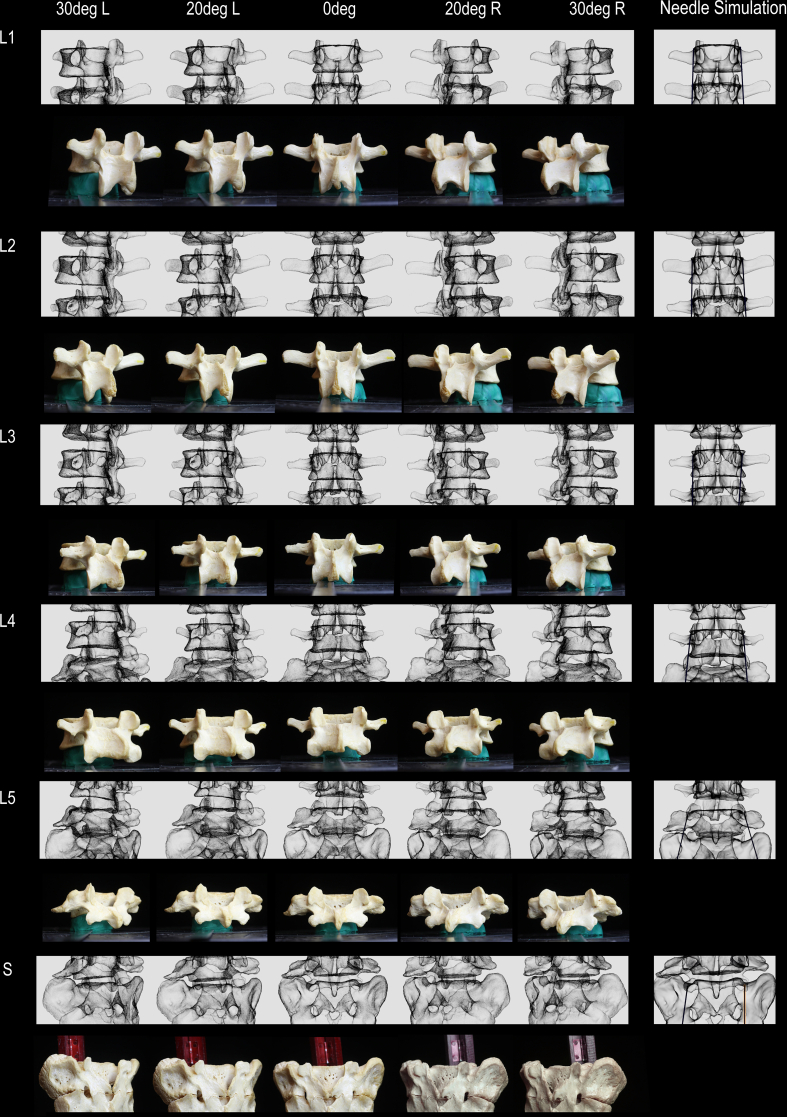


**figs5:**
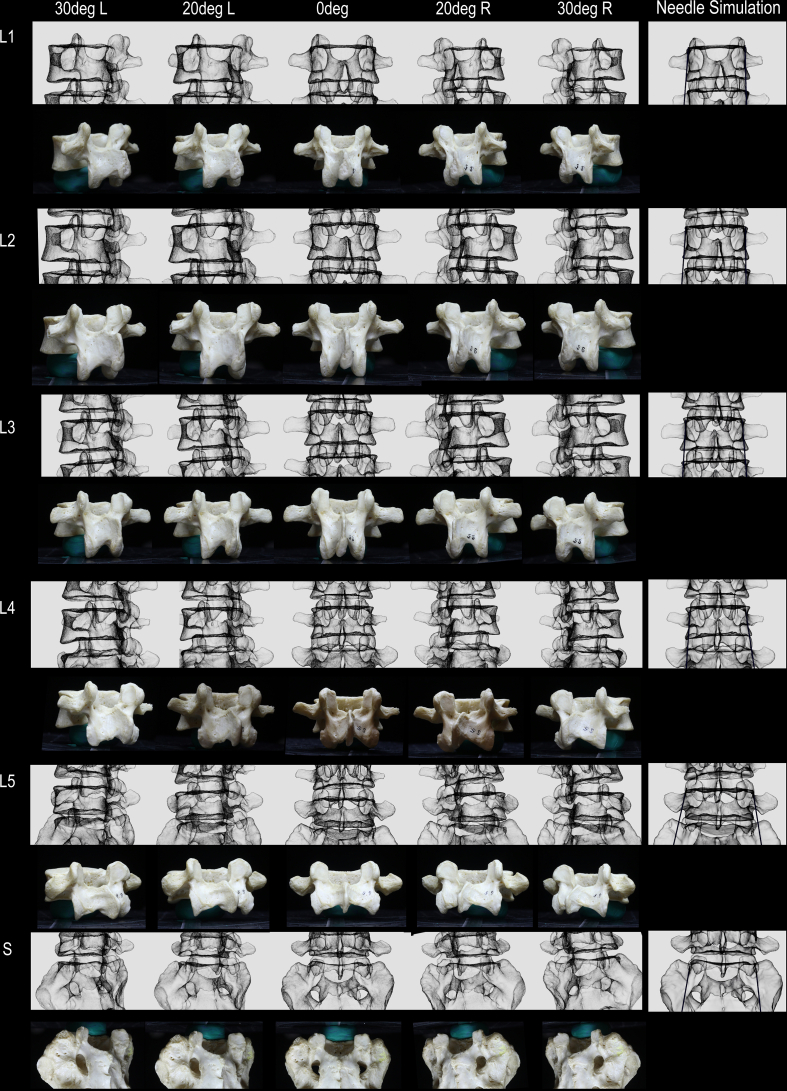


**figs6:**
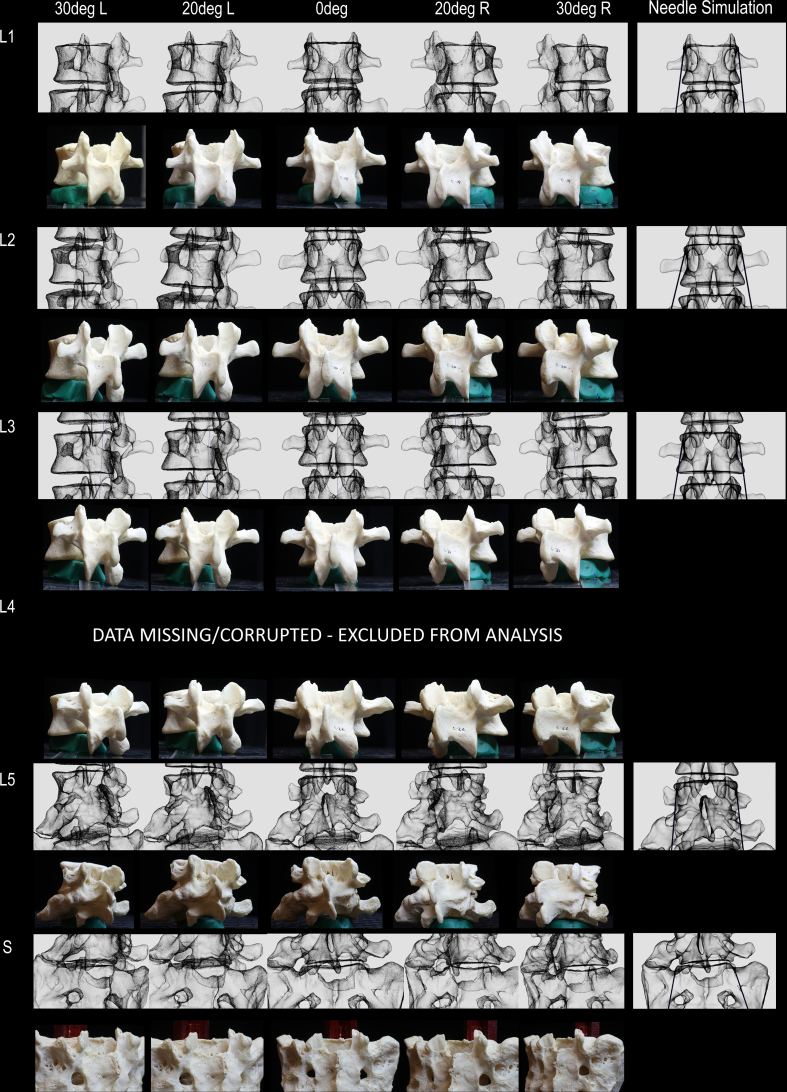


**figs7:**
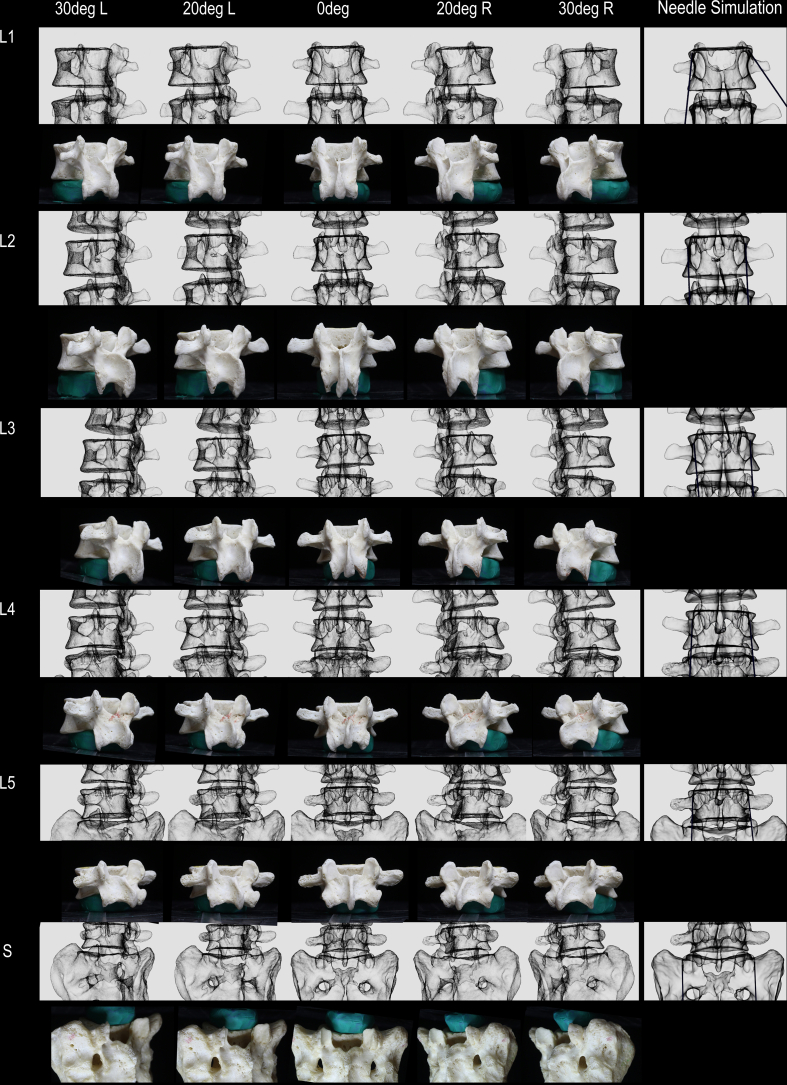


**figs8:**
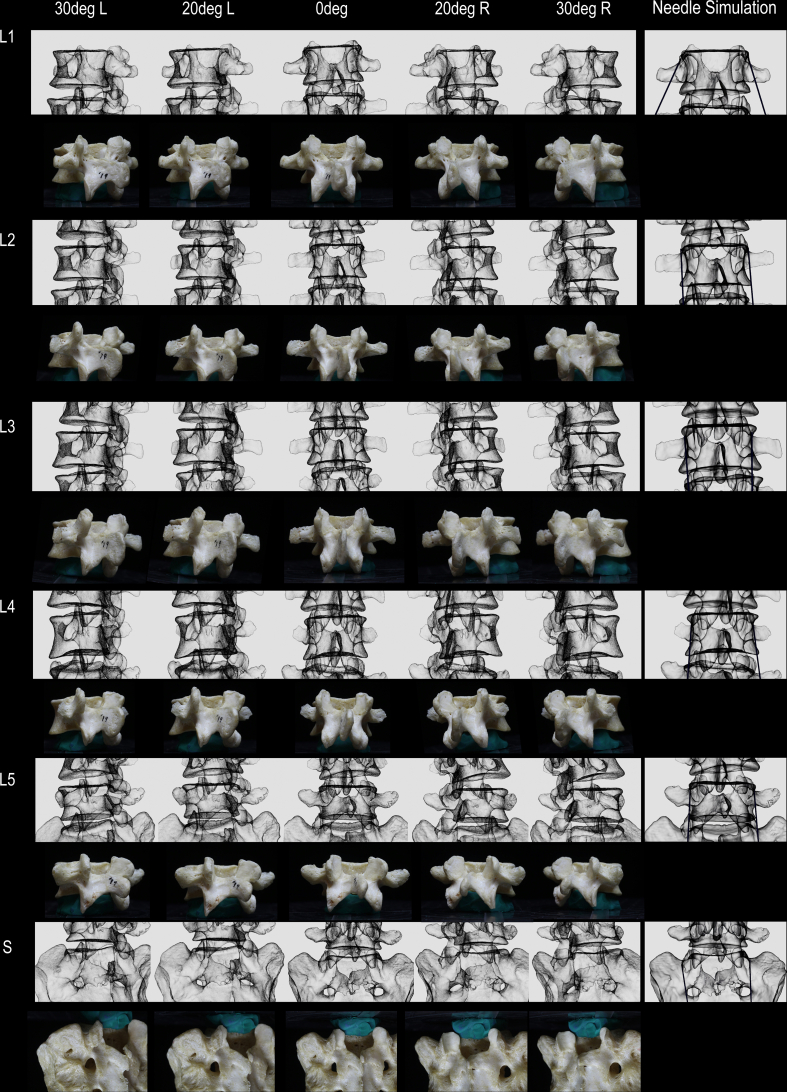


**figs9:**
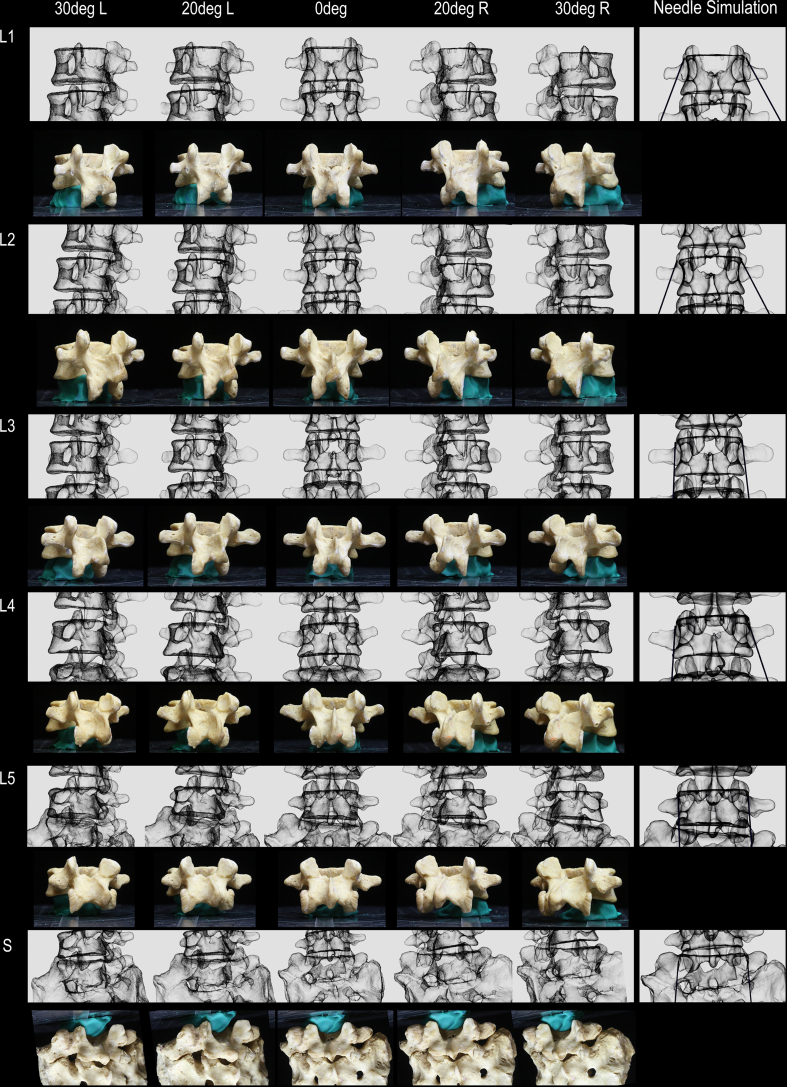


**figs10:**
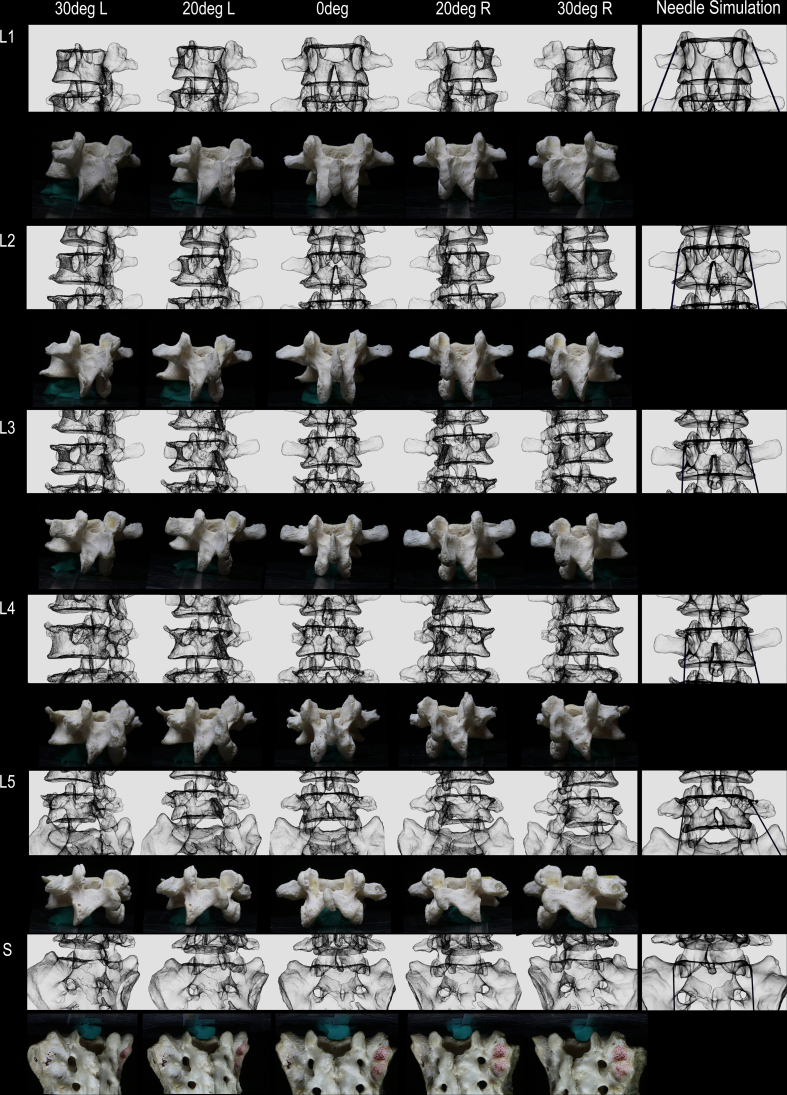


## References

[1] Schneider B.J., Doan L., Maes M.K., Martinez K.R., Gonzalez Cota A., Bogduk N. (2020). Systematic review of the effectiveness of lumbar medial branch thermal radiofrequency neurotomy, stratified for diagnostic methods and procedural technique. Pain Med.

[2] Zachariah C., Mayeux J., Alas G. (2020). Physiological and functional responses of water-cooled versus traditional radiofrequency ablation of peripheral nerves in rats. Reg Anesth Pain Med.

[3] Lau P., Mercer S., Govind J., Bogduk N. (2004). The surgical anatomy of lumbar medial branch neurotomy (facet denervation). Pain Med.

[4] Tran J., Peng P., Loh E. (May 19, 2022). Anatomical study of the medial branches of the lumbar dorsal rami: implications for image-guided intervention. Reg Anesth Pain Med.

[5] Jones-Whitehead C., Tran J., Wilson T.D., Loh E. (2025). Evaluation of a novel nerve ablation technique to relieve lower back pain: a cadaveric feasibility pilot study. Pain Med.

[6] Tran J., Lawson A., Agur A., Loh E. (2024). Parasagittal needle placement approach for lumbar medial branch denervation: a brief technical report. Reg Anesth Pain Med.

[7] Tran J., Campisi E.S., Agur A.M.R., Loh E. (2023). Quantification of needle angles for traditional lumbar medial branch radiofrequency ablation: an osteological study. Pain Med.

[8] Tran J., Campisi E.S., Agur A.M.R., Loh E. (2024 Jan 4). Quantification of needle angles for lumbar medial branch denervation targeting the posterior half of the superior articular process: an osteological study. Pain Med.

[9] Tran J., Campisi E., Roa Agudelo A., Agur A.M., Loh E. (2024). High-fidelity 3D modelling of the lumbar dorsal rami. Interv Pain Med.

[10] Tran J., Lawson A., Billias N., Loh E. (2024). 3D nerve proximity mapping of the medial branch of lumbar dorsal ramus: an anatomical study. Interv Pain Med.

[11] Tran J., Alboog A., Barua U., Billias N., Loh E. (2024). Optimal caudal needle angulation for lumbar medial branch denervation: a 3D cadaveric and clinical imaging comparison study. Interv Pain Med.

[12] Waring P.H., Rivers W.E., Bralts D.L. (2025). True AP imaging during lumbar medial branch radiofrequency neurotomy: interobserver reliability. Interv Pain Med.

[13] MacVicar J., Borowczyk J.M., MacVicar A.M., Loughnan B.M., Bogduk N. (2013). Lumbar medial branch radiofrequency neurotomy in New Zealand. Pain Med.

[14] Dreyfuss P., Halbrook B., Pauza K., Joshi A., McLarty J., Bogduk N. (2000). Efficacy and validity of radiofrequency neurotomy for chronic lumbar zygapophysial joint pain. Spine.

[15] Provenzano D.A., Holt B., Danko M. (2025). Assessment of real-world, prospective outcomes in patients treated with lumbar radiofrequency ablation for chronic pain (RAPID). Interv Pain Med.

[16] van Wijk R.M., Geurts J.W., Wynne H.J. (2005). Radiofrequency denervation of lumbar facet joints in the treatment of chronic low back pain: a randomized, double-blind, sham lesion-controlled trial. Clin J Pain.

[17] Conger A., Burnham T., Salazar F. (2020). The effectiveness of radiofrequency ablation of medial branch nerves for chronic lumbar facet joint syndrome in patients selected by guideline-concordant dual comparative medial branch blocks. Pain Med.

